# Improving *Salmonella *vector with *rec *mutation to stabilize the DNA cargoes

**DOI:** 10.1186/1471-2180-11-31

**Published:** 2011-02-08

**Authors:** Xiangmin Zhang, Soo-Young Wanda, Karen Brenneman, Wei Kong, Xin Zhang, Kenneth Roland, Roy Curtiss

**Affiliations:** 1 The Biodesign Institute, Arizona State University, Tempe, AZ 85287, USA; 2 School of Life Science, Arizona State University, Tempe, AZ 85287, USA; 3 Department of Biology, Washington University, St. Louis, MO 63130, USA; 4 Department of Pathology and Immunology, Washington University School of Medicine, 660 S. Euclid Avenue, St. Louis, MO 63110, USA

## Abstract

**Background:**

*Salmonella *has been employed to deliver therapeutic molecules against cancer and infectious diseases. As the carrier for target gene(s), the cargo plasmid should be stable in the bacterial vector. Plasmid recombination has been reduced in *E. coli *by mutating several genes including the *recA*, *recE*, *recF *and *recJ*. However, to our knowledge, there have been no published studies of the effect of these or any other genes that play a role in plasmid recombination in *Salmonella enterica*.

**Results:**

The effect of *recA*, *recF *and *recJ *deletions on DNA recombination was examined in three serotypes of *Salmonella enterica*. We found that (1) intraplasmid recombination between direct duplications was RecF-independent in Typhimurium and Paratyphi A, but could be significantly reduced in Typhi by a Δ*recA *or Δ*recF *mutation; (2) in all three *Salmonella *serotypes, both Δ*recA *and Δ*recF *mutations reduced intraplasmid recombination when a 1041 bp intervening sequence was present between the duplications; (3) Δ*recA *and Δ*recF *mutations resulted in lower frequencies of interplasmid recombination in Typhimurium and Paratyphi A, but not in Typhi; (4) in some cases, a Δ*recJ *mutation could reduce plasmid recombination but was less effective than Δ*recA *and Δ*recF *mutations. We also examined chromosome-related recombination. The frequencies of intrachromosomal recombination and plasmid integration into the chromosome were 2 and 3 logs lower than plasmid recombination frequencies in Rec^+ ^strains. A Δ*recA *mutation reduced both intrachromosomal recombination and plasmid integration frequencies.

**Conclusions:**

The Δ*recA *and Δ*recF *mutations can reduce plasmid recombination frequencies in *Salmonella enterica*, but the effect can vary between serovars. This information will be useful for developing *Salmonella *delivery vectors able to stably maintain plasmid cargoes for vaccine development and gene therapy.

## Background

Attenuated *Salmonella *are being developed as vaccines to protect against typhoid fever [1-3]. There are also endeavors employing *Salmonella *as delivery vectors for therapeutic molecules. One strategy utilizes attenuated *Salmonella*, which expresses a gene or gene fragment encoding a protective antigen as vaccine against bacterial pathogens [4-6]. The heterologous genes can be expressed from the *Salmonella *chromosome, or, more often, from a multi-copy plasmid [7,8]. Another strategy exploits *Salmonella *as a delivery vector of DNA vaccine against viral pathogens [4,5,9]. The later strategy is also used to deliver DNA encoding tumor antigen or cytokine for therapeutic applications in oncology [10,11]. In addition, *Salmonella *is used to deliver small interfering RNAs (siRNA) [12], ribozymes [13] and large DNA molecules encoding a viral genome [14]. For instance, *in vivo *delivery of an artificial bacterial chromosome (BAC) carrying the viral genome of the murine cytomegalovrirus (MCMV) by *Salmonella *Typhimurium led to a productive virus infection in mice and resulted in elevated titers of specific antibodies against lethal MCMV challenge [14].

Most vaccine designs utilize *Salmonella *delivery vectors carrying a single plasmid for expression of a single antigen or of a fusion protein carrying epitopes from more than one antigen [15]. To induce broader immunity against a particular pathogen or various pathogens, one might need to express multiple antigens from a single plasmid carrying different antigen cassettes or from multiple plasmids in a single cell, each expressing one or more relevant antigens. Co-delivery of plasmids encoding tumor antigens and cytokines by *Salmonella *has been successfully demonstrated to improve protective immunity against cancer [16]. In the case where multiple plasmids are carried in the same *Salmonella *vector strain, there are most likely regions of homology between the plasmids, since the widely used pUC- and pBR-based plasmids have origins of replication that are nearly identical and both share regions of homology with the p15A *ori*. Additionally, commonly used promoter sequences, transcriptonal terminators and other expression plasmid components may also be present on plasmids coexisting in the same bacterial cell. The presence of these similar or identical DNA sequences would serve to facilitate undesirable interplasmid recombination. In some cases the bacterial vector may intentionally harbor multiple copies of the same DNA sequence, which may lead to plasmid instability. Recently, we encountered such a situation during the development of a bacterial based influenza vaccine. We constructed a single plasmid carrying eight head-to-tail connected influenza cDNA cassettes [17]. The plasmid was intended for delivery into host cells by an attenuated *Salmonella *strain. The multiple repetitive sequences residing in the plasmid make its stability within the attenuated *Salmonella *an important concern because any intraplasmid recombination event results in deletion of one or more influenza gene cassettes.

Recent work in our laboratory has focused on developing new strategies for attenuated *Salmonella *vaccine strains, with features including regulated delayed *in vivo *attenuation [18,19], regulated delayed *in vivo *antigen synthesis [18,20-22], and programmed delayed *in vivo *cell lysis [23,24]. For all of these systems, one or more chromosomal and/or plasmid genes are placed under the control of the *araC *P_BAD _promoter. Eventually, our goal is to combine all of these features into a single *Salmonella *vaccine vector strain. Such a strain will therefore carry multiple chromosomal and plasmid copies of *araC *P_BAD_, providing sites for potential recombination, which could lead to unwanted chromosomal or plasmid rearrangements.

However, to our knowledge, there have been no published studies specifically designed to evaluate plasmid recombination in *Salmonella enterica*. Deletions of several *Escherichia coli *genes are known to reduce the frequency of plasmid recombination, including the *recA*, *recE*, *recF *and *recJ *genes [25-30]. The *recA *gene encodes the general recombinase RecA, involved in nearly all forms of recombination in the cell [31]. The RecE, RecF and RecJ proteins play a role in plasmid recombination and recombination repair [32,33]. The RecA, RecF and RecJ proteins are highly homologous between *E. coli *and *S. enterica*, therefore they may play similar roles in DNA recombination. Despite these possible similarities, the recombination systems in the two organisms differ somewhat, as *S. enterica *does not encode *recE *[34]. Based on these concerns, we decided to determine the effect of *rec *gene deletions on intraplasmid recombination, interplasmid recombination, intrachromosomal recombination and plasmid integration in *S. enterica*.

In this work, we examine the effect of Δ*recA*, Δ*recF* and Δ*recJ* mutations on DNA recombination frequencies in three serovars of *Salmonella enterica *currently relevant to vaccine development. Our results show that the effect of these mutations on recombination can vary among *Salmonella *serovars and with previously published results in *E. coli*.

## Results

### Plasmid construction

**Figure 1:**
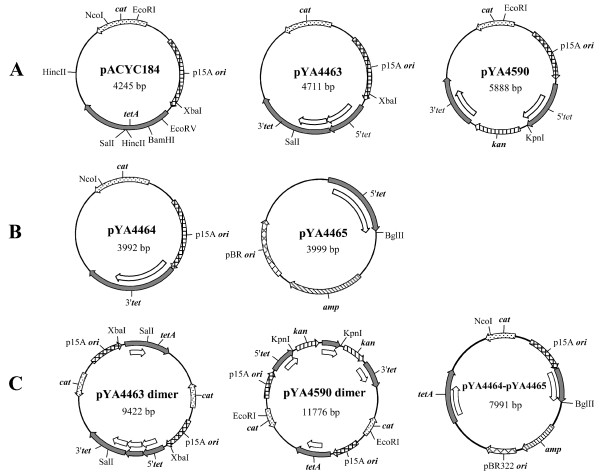
**Illustration of plasmids carrying intact or truncated *tetA *genes**. Plasmids are not drawn to scale. (A) Plasmid pACYC184 carries an intact *tetA *gene (1191 bp), which is the source of all truncated *tetA *genes used in this study. Plasmid pYA4463 carries two copies of truncated *tetA *genes, truncated at the 5' or 3' ends as indicated, which results in a 466-bp direct tandem duplication (shown as open arrows). Plasmid pYA4590 has two similar copies of truncated *tetA *genes, resulting in 602 bp of repetitive sequence (shown as open arrows) separated by 1041-bp *kan *cassette. (B) Plasmid pYA4464 has a 3'*tet *truncated gene. Plasmid pYA4465 has a 5'*tet *truncated gene. There are 751 bp of common sequences (shown as open arrows) between the two truncated *tetA *genes. (C) Plasmid pYA4463 dimer is the intermolecular recombination product of two pYA4463 molecules. Plasmid pYA4590 dimer is the intermolecular recombination product of two pYA4590 molecules. Plasmid pYA4464-pYA4465 is the intermolecular recombination product of pYA4464 and pYA4465.

**Figure 2:**
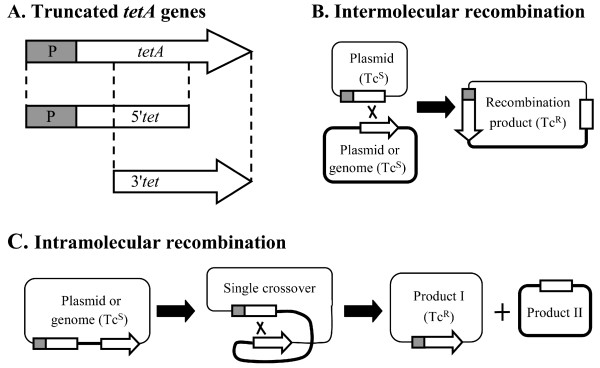
**Strategies for measuring DNA recombination**. (A) Truncated *tetA *genes. Two truncated *tetA *genes were derived from an intact *tetA *gene and its promoter (P). 5'*tet*, includes the *tetA *promoter and the 5' portion of *tetA *gene. 3'*tet*, consists of the 3' portion of the *tetA *gene. The overlapping region (between 5'*tet *and 3'*tet*) varies from 466 to 789 bp depending on the system. Homologous recombination can occur between the two truncated *tetA *genes at the overlapping region, leading to the formation of a functional *tetA *gene. (B) Intermolecular recombination. Each DNA molecule carries either 5'*tet *or 3'*tet*. A single crossover between the two molecules occurs at the regions of homology, and leads to a functional *tetA *gene. (C) Intramolecular recombination. The two truncated *tetA *genes were placed on one molecule in the same orientation. A single crossover between the regions of homology leads to a functional *tetA *gene.

We constructed a series of plasmids (Figure 1 and Table 1) encoding various truncated *tetA *genes to assay plasmid recombination frequencies using the strategies similar to those described previously [28,35]. Restoration of a functional *tetA *gene via intra- or intermolecular recombination resulted in a change of the bacterial phenotype from tetracycline sensitive to tetracycline resistant, and served as a marker allowing us to measure the frequency of recombination events (Figure 2).

**Table 1:** Plasmids used in this study

Plasmid	Relevant characteristic(s)*	Reference or source
pACYC184	*cat*, *tetA*, p15A *ori*	[59]
pBAD-HisA	*amp*, pBR *ori*	Invitrogen
pKD46	λ Red recombinase expression plasmid	[60]
p15A-PB2-kan	*cat*, *kan*, p15A *ori*	This study
pYA4463	pACYC184, adjacent 5'*tet *and 3'*tet*	This study
pYA4464	pACYC184, 3'*tet*	This study
pYA4465	pBAD-HisA; 5'*tet*	This study
pYA4590	pACYC184, 5'*tet*-*kan*-3'*tet*	This study
pYA4373	*cat*-*sacB*	[54]
pRE112	*ori*T, *ori*V, *sacB*, *cat*	[61]
pYA3886	pRE112, Δ*recF126*	This study
pYA4783	pYA3886, Δ*recF1074*	This study
pYA3887	pRE112, Δ*recJ1315*	This study
pYA4680	pRE112, Δ*recA62*	This study
pYA4518	pYA4464, *cat*, p15A *ori*, GFP gene	This study
pYA4518-cysG	Two *cysG *fragments	This study
pYA4689	pYA4518-cysG, 5'*tet-kan-*3'*tet*	This study
pYA4690	pYA4518-cysG, 5'*tet-kan*	This study
pYA5001	*aacC1*, pSC101 *ori*, T vector	This study
pYA5002	pYA5001, *recA *cassette from Typhimurium χ3761	This study
pYA5004	pYA5001, *recA *cassette from Typhi Ty2 χ3769	This study
pYA5005	pYA5001, *recF *gene from Typhimurium χ3761	This study
pYA5006	pYA5001, *recF *gene from Typhi Ty2 χ3769	This study

* *cat*: chloramphenicol resistance gene; *tetA*: tetracycline resistance gene; *amp*: ampicillin resistance gene; *kan*: kanamycin resistance gene; 3'*tet*: 3' portion of the *tetA *gene; 5'*tet*: 5' portion of the *tetA *gene together with its promoter; *aacC1*: 3-N-aminoglycoside acetyltransferase.

Plasmids pYA4463 and pYA4590 were constructed to test intraplasmid recombination (Figure 1 panel A). Plasmid pYA4463 carries two truncated *tetA *genes (5' end and 3'end), which have 466-bp of tandemly repeated sequence. An intramolecular recombination event can delete one of the repeats resulting in an intact *tetA *gene, thereby recreating the structure of plasmid pACYC184 (Figure 1 panel A). Theoretically, intermolecular recombination may occur between two pYA4463 molecules to form a plasmid dimer with a functional *tetA *gene (Figure 1 panel C). Plasmid pYA4590 contains a 602-bp *tetA *sequence duplication separated by a 1041-bp *kan *cassette. The intramolecular recombination product is equivalent to pACYC184. The intermolecular recombination product is a dimer plasmid containing an intact *tetA *gene (Figure 1 panel C). Plasmids pYA4464 and pYA4465 carry the 3'*tet *gene and 5'*tet *gene, respectively (Figure 1). The Rec^+ ^*Salmonella *strain χ3761 carrying either plasmid individually was sensitive to tetracycline. There is 751-bp of *tetA *DNA in common between the two truncated *tetA *genes. Recombination between the two plasmids creates a hybrid plasmid containing an intact *tetA *gene (Figure 1 panel C).

### Intraplasmid recombination products

**Figure 3:**
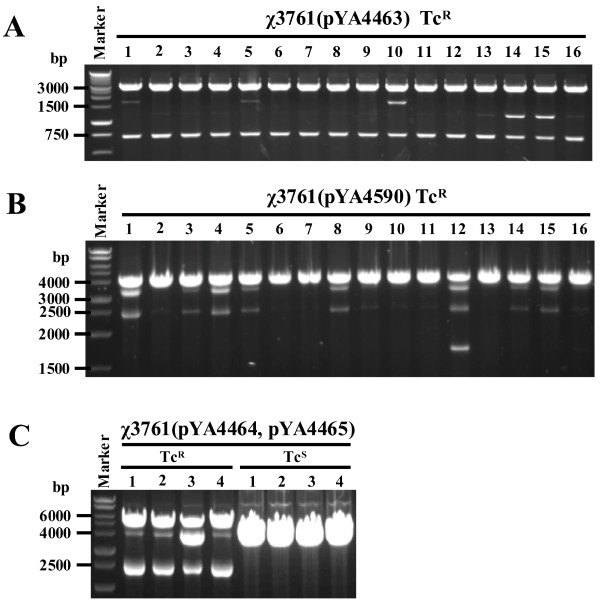
**Verification of plasmid recombination product by agarose gel separation**. (A) Plasmid DNA was isolated from Tc^R ^clones derived from χ3761(pYA4463) and digested by *Xba*I and *Sal*I. (B) Plasmid DNA was isolated from Tc^R ^clones of χ3761(pYA4590) and digested by *Kpn*I and *Eco*RI. (C) Plasmid DNA was isolated from Tc^R ^or Tc^S ^clones of χ3761(pYA4464, pYA4465). The purified plasmids were digested with *Nco*I and *Bgl*II.

To verify the recombination products, plasmid DNA was prepared from tetracycline resistant (Tc^R^) single colonies derived from χ3761(pYA4463), χ3761(pYA4590) and χ3761(pYA4464, pYA4465). Plasmids extracted from Tc^R ^clones of χ3761(pYA4463) were digested with *Xba*I and *Sal*I. Theoretically, *Xba*I/*Sal*I digestion of pYA4463 will yield two fragments (3524 bp and 1187 bp), pACYC184 will yield two fragments (3524 bp and 721 bp) and pYA4463 dimer will yield four fragments (3524 bp, 3524 bp, 1653 bp and 721 bp). The results (Figure 3A) showed that digestion of all 16 Tc^R ^clones yielded a 721-bp band, indicating either a pYA4463 dimer or a plasmid equivalent to pACYC184. Three clones (lane 1, 5 and 10) yielded the pYA4463 dimer-specific 1653-bp band. Therefore, we conclude that the other 13 clones recombined to form the pACYC184-like structure. Of note, several clones (2, 13-16) also yielded the 1187-bp pYA4463-specific band, suggesting that the original plasmid (pYA4463) and its recombination product (pACYC184-like) could coexist in the same bacterial cell.

Plasmids extracted from Tc^R ^clones of χ3761(pYA4590) were digested with *Kpn*I and *Eco*RI. Theoretically, plasmid pYA4590 will be digested into two fragments (3414 bp and 2474 bp), plasmid pACYC184 will be linearized (4245 bp) and the pYA4590 plasmid dimer will be digested into four fragments (4245 bp, 3414 bp, 2474 bp and 1643 bp). Examination of the restricted DNA (Figure 3B) showed that only one clone (lane 12) had the pYA4590 dimer-specific 1643-bp band. The most prominent band in the other lanes was a 4245-bp band expected for pACYC184-like recombination products. Nine clones contained a mixture of pACYC184 and pYA4590 (lane 1, 3-5, 8, 9, 14-16).

### Interplasmid recombination products

Plasmids extracted from Tc^R ^clones of χ3761(pYA4464, pYA4465) were digested with *Nco*I and *Bgl*II. Both pYA4464 and pYA4465 are linearized into a DNA fragment about 4 kb. Therefore, in cells containing each or both monomeric plasmids, the digested product will be a single band. The pYA4464-pYA4465 hybrid will be cut into two fragments (5510 bp and 2481 bp). All four of the Tc^R ^clones we isolated and examined showed recombination product specific bands and the 4-kb band expected when each plasmid exists separately in the cell. Four tetracycline sensitive (Tc^S^) isolates were examined and only a single band was observed, as expected (Figure 3C). These results suggest that interplasmid recombination occurred in the Tc^R ^cells and that both dimer and individual monomers corresponding to at least one of the two starting plasmids can coexist in the same bacterial cell. We performed a similar experiment in *S*. Typhi strain Ty2(pYA4464, pYA4465) and obtained identical results (data not shown).

### Construction of *rec *deletion strains

**Figure 4:**
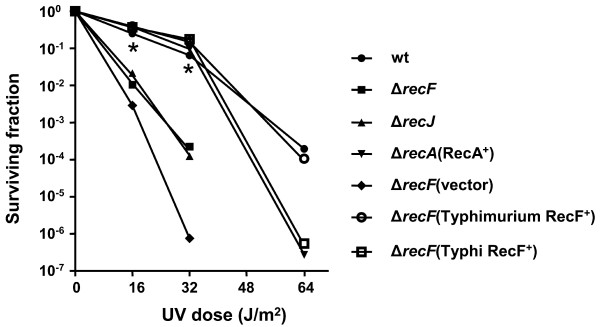
**UV sensitivity of *S*. Typhimurium *rec *mutants**. Log phase cultures of *S*. Typhimurium were diluted and spread on LB agar. Multiple dilutions were exposed to 254 nm UV in a dark room at each designated dose. Then the plates were wrapped with aluminum foil and placed at 37°C overnight. Surviving fractions were calculated and shown except Δ*recA* strains χ9833 and χ9833(pYA5001), for which no survivors were recovered at any UV dose. wt: χ3761; Δ*recF*: χ9070; Δ*recJ*: χ9072; Δ*recA*(RecA^+^): χ9833(pYA5002); Δ*recF*(vector): χ9070(pYA5001); Δ*recF*(Typhimurium RecF^+^): χ9070(pYA5005); Δ*recF*(Typhi RecF^+^): χ9070(pYA5006). Survival of Rec^+ ^strains [χ3761, χ9833(pYA5002), χ9070(pYA5005) and χ9070(pYA5006)] was significantly greater than survival of the Rec^- ^strains [χ9070, χ9072 and χ9070(pYA5001)] at the UV doses indicated (*P *≤ 0.002; *).

We constructed a series of strains for these studies carrying deletions in either *recA*, *recF *or *recJ *in *S. *Typhimurium UK-1, *S*. Typhi Ty2 and *S*. Paratyphi A (Table 2). We also constructed Δ*recA*Δ *recF* and Δ*recJ* Δ *recF* double mutants in *S. *Typhimurium. Deletion of *recA, recF *and *recJ *results in an increase in sensitivity to UV irradiation [36,37]. To verify the presence of these deletions phenotypically in our strains, the UV sensitivity of the *S*. Typhimurium mutant strains was measured. The Δ*recF *and Δ*recJ *mutants showed significantly lower surviving fractions than the wild type strain after the same exposure dose (Figure 4). By contrast, after five seconds of UV exposure (16 J/m^2^) to 2.2 × 10^9 ^CFU of the Δ*recA62 *mutant (χ9833), we were unable to recover any surviving cells (not shown). UV resistance similar to the wild-type strain χ3761 was restored to *S*. Typhimurium Δ*recA *and Δ*recF *mutants strains after introduction of *recA *plasmid (pYA5002) or either *recF *plasmid (pYA5005/pYA5006), respectively. Transformation of either mutant strain with vector plasmid pYA5001 did not restore UV resistance (Figure 4 and data not shown for *recA *mutant).

**Table 2:** The bacterial strains used in this study

Strain	Genotype* [parental strain]	Reference or source
*S*. Typhimurium UK-1		
χ3761	wild type	[62]
χ9833	Δ*recA62 *[χ3761]	This study
χ9070	Δ*recF126 *[χ3761]	This study
χ9072	Δ*recJ1315 *[χ3761]	This study
χ9081	Δ*recJ1315 *Δ*recF126 *[χ9072]	This study
χ9931	*cysG494*::(5'*tet*-*kan*-3'*tet*) [χ3761]	This study
χ9932	Δ*recF126 cysG494*::(5'*tet*-*kan*-3'*tet*) [χ9070]	This study
χ9933	Δ*recJ1315 cysG494*::(5'*tet*-*kan*-3'*tet*) [χ9072]	This study
χ9934	Δ*recA62 cysG494*::(5'*tet*-*kan*-3'*tet*) [χ9833]	This study
χ9935	*cysG493*::(5'*tet*-*kan*) [χ3761]	This study
χ9936	Δ*recF126 cysG493*::(5'*tet*-*kan*) [χ9070]	This study
χ9937	Δ*recJ1315 cysG493*::(5'*tet*-*kan*) [χ9072]	This study
χ9938	Δ*recA62 cysG493*::(5'*tet*-*kan*) [χ9833]	This study
χ9939	Δ*recF126 Δ recA62 *[χ9070]	This study
*S*. Typhi Ty2		
χ3769	wild type	[63]
χ11053	Δ*recF126 *[χ3769]	This study
χ11134	Δ*recF1074 *[χ3769]	This study
χ11159	Δ*recA62 *[χ3769]	This study
χ11194	Δ*recJ1315 *[χ3769]	This study
*S*. Typhi ISP1820		
χ3744	wild type	D.M. Hone
χ11133	Δ*recF1074 *[χ3744]	This study
*S*. Paratyphi A		
χ8387	Plasmid pSPA1 was cured from wt isolate ATCC 9281	This study
χ11243	Δ*recA62 *[χ8387]	This study
χ11244	Δ*recF126 *[χ8387]	This study
χ11245	Δ*recJ1315 *[χ8387]	This study
*E. coli *K-12		
EPI300	F^- ^*mcrA *Δ* (mrr-hsdRMS-mcrBC) *Φ80d*lacZ *Δ* M15 *Δ* lacX74 recA1 endA1 araD139 *Δ* (ara, leu)7697 galU galK λ*^- ^*rpsL nupG trfA dhfr*	Epicentre
χ7213 (MGN-617)	*thi-1 thr-1 leuB6 glnV44 fhuA21 lacY1 recA1 RP4-2-Tc*::Muλ*pir *Δ*asdA4 *Δ*zhf-2*::Tn10	[55]

* *kan*: kanamycin resistance gene; 5'*tet*: 5' portion of the *tetA *gene together with its promoter; 3'*tet*: 3' portion of the *tetA *gene.

### Effect of *rec *deletions on intraplasmid recombination

To examine the influence of Δ*recA*, Δ*recF *and Δ*recJ *mutations on intraplasmid recombination frequencies, plasmid pYA4463 (tandem duplication) or pYA4590 (tandem duplication with intervening sequence) were introduced into *Salmonella rec *mutants and their parental strains and analyzed as described in the Methods section. The recombination frequency of plasmid pYA4463 was approximately 1.5-5.0 × 10^-3 ^in Rec^+ ^Typhimurium, Typhi and Paratyphi A (Table 3). In *S. *Typhimurium and Paratyphi A, most of the *rec *deletions had no effect on the intraplasmid recombination frequency of plasmid pYA4463 except that a small, but statistically significant decrease in recombination was observed in the Δ*recA *mutant of Paratyphi A. However, in both *S. *Typhi strains, both Δ*recF *mutations resulted in approximately 10-fold decrease in recombination frequency (*P *< 0.01), while the Δ*recA *and Δ*recJ *mutations resulted in a 2-3-fold reduction (*P *< 0.01). In the complementation test, the recombination frequency of plasmid pYA4463 in *S*. Typhi χ11053 was restored to 2.52 ± 0.18 × 10^-3 ^and 1.71 ± 0.68 × 10^-3 ^by introduction of plasmid pYA5005 encoding *S*. Typhimurium *recF *gene and pYA5006 encoding the *S*. Typhi *recF *gene, respectively (Table 3).

**Table 3:** Plasmid recombination frequency (Mean ± STD, × 10^-^^3^)

Strain	*rec *deletion	pYA4463^a^	pYA4590^b^	pYA4464+pYA4465^c^
*S*. Typhimurium				
χ3761	None	1.55 ± 0.31	2.40 ± 0.54	2.88 ± 0.85
χ9833	Δ*recA62*	1.07 ± 0.24	0.22 ± 0.07**	0.27 ± 0.07**
χ9070	Δ*recF126*	1.14 ± 0.15	0.52 ± 0.07**	0.33 ± 0.09**
χ9072	Δ*recJ1315*	1.87 ± 0.44	2.37 ± 0.21	1.10 ± 0.20**
χ9081	Δ*recJ1315 *Δ*recF126*	NA^d^	NA	0.35 ± 0.08**
χ9939	Δ*recF126 *Δ*recA62*	NA	0.41 ± 0.09**	0.35 ± 0.08**
χ9833(pYA5002)	Δ*recA62 *(RecA^+^)	NA	2.50 ± 0.42	NA
χ9070(pYA5005)	Δ*recF126 *(RecF^+^)	NA	2.00 ± 0.24	NA
*S*. Typhi Ty2				
χ3769	None	4.69 ± 0.26	11.59 ± 2.61	4.20 ± 1.44
χ11159	Δ*recA62*	1.32 ± 0.27**	0.60 ± 0.19**	3.37 ± 0.96
χ11053	Δ*recF126*	0.51 ± 0.06**	0.57 ± 0.09**	6.19 ± 2.71
χ11134	Δ*recF1074*	0.45 ± 0.05**	0.52 ± 0.17**	16.28 ± 2.64**
χ11194	Δ*recJ1315*	1.69 ± 0.26**	4.88 ± 1.56**	2.31 ± 0.90
χ11053(pYA5005)	Δ*recF126 *(RecF^+^)	2.52 ± 0.18	NA	NA
χ11053(pYA5006)	Δ*recF126 *(RecF^+^)	1.71 ± 0.68	NA	NA
χ11159(pYA5002)	Δ*recA62 *(RecA^+^)	NA	14.35 ± 2.44	NA
χ11053(pYA5006)	Δ*recF126 *(RecF^+^)	NA	2.86 ± 0.59	NA
*S*. Typhi ISP1820				
χ3744	None	4.93 ± 0.67	13.10 ± 1.23	4.22 ± 0.25
χ11133	Δ*recF1074*	0.65 ± 0.26**	0.71 ± 0.06**	5.38 ± 0.58
*S*. Paratyphi A				
χ8387	None	2.70 ± 0.39	3.32 ± 0.61	1.03 ± 0.15
χ11243	Δ*recA62*	1.91 ± 0.69**	0.55 ± 0.20**	0.13 ± 0.03**
χ11244	Δ*recF126*	5.00 ± 0.70	1.16 ± 0.21**	0.34 ± 0.04**
χ11245	Δ*recJ1315*	2.56 ± 0.41	1.83 ± 0.99**	0.64 ± 0.15**

^a^Intraplasmid recombination without intervening sequence (5'*tet*-3'*tet*).

^b ^Intraplasmid recombination with a 1041-bp intervening sequence (5'*tet*-*kan*-3'*tet*).

^c ^Interplasmid recombination.

^d ^Not assayed.

** *P *< 0.01, relative to the parental *rec*^+ ^strain.

The results with plasmid pYA4590 were also variable among strains. The recombination frequency in Rec^+ ^*S. *Typhimurium and *S*. Paratyphi A strains was approximately 2-3 × 10^-3 ^and in both *S. *Typhi strains, the frequency was 3-fold higher, at 1.16 × 10^-2 ^(Ty2) and 1.31 × 10^-2 ^(ISP1820). In *S. *Typhimurium and *S. *Typhi Ty2, the Δ *recA* and Δ*recF* mutations reduced the recombination frequency of plasmid pYA4590 by 5-20-fold (*P *< 0.01; Table 3). The results were similar for *S*. Paratyphi A, though the Δ*recF* mutation only led to 3-fold lower plasmid pYA4590 recombination (*P *< 0.01). The Δ*recJ* mutation had no effect in *S*. Typhimurium and resulted in a 2-3-fold decrease in recombination in both *S. *Typhi Ty2 and *S*. Paratyphi A. Combining the Δ*recA* Δ*recF* mutations in *S. *Typhimurium led to a recombination frequency similar to the frequencies observed for both mutations individually, indicating no additive effect. In the complementation test, plasmid pYA5002, which encodes *S*. Typhimurium *recA*, was transformed into *S*. Typhimurium Δ*recA *mutant χ9833(pYA4590) and *S*. Typhi Δ*recA *mutant χ11159(pYA4590). Their respective recombination frequencies were 2.50 ± 0.42 × 10^-3 ^and 14.35 ± 2.44 × 10^-3^, which were comparable to the corresponding wild type strains (*P *> 0.05) (Table 3). The *recF*-encoding plasmids pYA5005 and pYA5006 were transformed into *recF *mutant strains χ9070(pYA4590) and χ11053(pYA4590), respectively. The respective recombination frequencies were increased to 2.00 ± 0.24 × 10^-3 ^and 2.86 ± 0.59 × 10^-3^.

### Effect of *rec *deletions on interplasmid recombination

To evaluate interplasmid recombination, plasmids pYA4464 and pYA4465 were co-electroporated into the wild-type and *rec *deletion strains. Electroporants from each test strain were grown in LB broth containing both ampicillin and chloramphenicol to maintain selection for both plasmids. The frequency of recombination was determined as described in the Methods section. The interplasmid recombination frequency was 1-4 × 10^-3 ^for Rec^+ ^*S*. Typhimurium, *S*. Typhi and *S*. Paratyphi A strains (Table 3). For Typhimurium and Paratyphi A, the Δ*recA *and each Δ*recF *mutation reduced the interplasmid recombination frequency by about 3-10-fold (*P *< 0.01). In contrast, the Δ*recA *mutation had no effect on interplasmid recombination in *S. *Typhi Ty2. The Δ*recF *mutations did not reduce interplasmid recombination in either of the Typhi strains. Surprisingly, introduction of the Δ*recF1074 *mutation into *S. *Typhi Ty2 resulted in significantly higher interplasmid recombination (*P *< 0.01). Note that we performed this analysis in eight independent experiments and observed a higher recombination frequency of interplasmid recombination each time. The Δ*recJ* mutation had no significant effect in *S. *Typhi, and a small (< 3-fold) but significant effect in *S. *Typhimurium and *S*. Paratyphi A. The recombination frequencies were also determined in *S*. Typhimurium strains Δ*recA* Δ*recF* and Δ*recF* Δ*recJ* double deletions. No additive effect between the two mutations was observed with respect to each single mutation.

### Effect of *rec *deletions on chromosome related recombination

To measure intrachomosomal recombination frequencies, we introduced the pYA4590-derived DNA sequence containing two truncated *tetA *genes (5'*tet-kan*-3'*tet*) into the *S. *Typhimurium chromosome at *cysG*. The two truncated *tetA *genes had 602 bp of overlapping sequence. Intrachromosomal recombination deletes the kanamycin resistance cassette and restores one intact copy of the *tetA *gene (Figure 2C). Deletion of *recA *resulted in a 5-fold reduced recombination frequency compared to the Rec^+ ^strain χ9931 (*P *< 0.01), while the *recF *or *recJ *deletions had no effect, indicating that RecF and RecJ are not involved in this process (Table 4).

**Table 4:** Chromosome related recombination in *S*. Typhimurium^a^

*rec *deletion	Intrachromosomal recombination		Plasmid integration	
		
	Strain	Frequency (10^-5^)	Strain	Frequency (10^-6^)
None	χ9931	6.02 ± 0.38	χ9935	5.59 ± 0.94
Δ*recF126*	χ9932	7.05 ± 1.40	χ9936	2.13 ± 0.60**
Δ*recJ1315*	χ9933	9.18 ± 2.18	χ9937	4.89 ± 0.41
Δ*recA62*	χ9934	1.29 ± 0.51**	χ9938^b^	<0.00071**

^a ^Mean ± STD from 3-5 assays were shown in the table.

^b ^Upon introduction of pKD46 (30°C, 0.2% arabinose), the frequency was 6.41 ± 0.85 × 10^-6 ^(*P *= 0.425).

** *P *< 0.01, relative to the parental *rec*^+ ^strain.

To examine plasmid integration, the 5'*tet *gene was introduced into the *S. *Typhimurium chromosome at *cysG*. The resulting strains were transformed with plasmid pYA4464 (3'*tet*) (Figure 1B). The 789 bp of overlapping sequence between 5'*tet *on the chromosome and the 3'*tet *on the plasmid could result in plasmid integration into the chromosome, generating an intact *tetA *gene (Figure 2B). Deletion of *recA *had a profound effect, reducing the integration frequency to less than 7 × 10^-10^, which was below the limits of detection in this assay (*P *< 0.01), indicating a strict requirement for RecA in this process. Introduction of plasmid pKD46, which encodes the λ Red recombinase, into χ9938 (Δ*recA*) carrying pYA4464 restored the integration frequency to the level of the Rec^+ ^strain χ9935. Deletion of *recF *reduced the frequency of integration less than 3-fold (*P *< 0.01; Table 4) and the Δ*recJ *deletion had no effect.

### Effect of *rec *deletions on the virulence of *S*. Typhimurium

BALB/c mice were orally inoculated with the highly virulent *S*. Typhimurium strain χ3761 and its *rec *mutant derivatives. The LD_50_s of χ3761, χ9070 (Δ*recF*) and χ9072 (Δ*recJ*) were similar, 3.2 × 10^4^, 6.8 × 10^4 ^and 1.5 × 10^5 ^CFU, respectively (Table 5). The LD_50 _of the Δ*recF* Δ*recJ *double mutant was approximately 100-fold higher than χ3761, at 2.2 × 10^6 ^CFU. All mice inoculated with 1.3 × 10^9 ^CFU of the Δ*recA *mutant survived, indicating that the LD_50 _was > 1.3 × 10^9 ^CFU. Two months following the initial inoculation with the Δ *recA *mutant strain, surviving mice were challenged with either 1.5 × 10^8 ^or 1.5 × 10^9 ^CFU of wild-type strain χ3761. All mice survived the challenge, indicating that Δ *recA *mutant strain χ9833 was both attenuated and immunogenic.

**Table 5:** Virulence of *S*. Typhimurium *rec *mutants in BALB/c mice (oral inoculation)

Strain	*rec *deletion	Dose (CFU)	Survivor/total	LD_50 _(CFU)
χ3761	None	1.5 × 10^6^	0/4	3.2 × 10^4^
		1.5 × 10^5^	1/4	
		1.5 × 10^4^	3/4	
		1.5 × 10^3^	4/4	
χ9070	Δ*recF126*	1.0 × 10^7^	0/4	6.8 × 10^4^
		1.0 × 10^6^	1/4	
		1.0 × 10^5^	1/4	
		1.0 × 10^4^	4/4	
χ9072	Δ*recJ1315*	1.0 × 10^7^	0/4	1.5 × 10^5^
		1.0 × 10^6^	0/4	
		1.0 × 10^5^	3/4	
		1.0 × 10^4^	3/4	
χ9081	Δ*recJ1315 Δ recF126*	1.0 × 10^7^	1/4	2.2 × 10^6^
		1.0 × 10^6^	3/4	
		1.0 × 10^5^	4/4	
		1.0 × 10^4^	3/4	
χ9833	Δ*recA62*	1.3 × 10^9^	10/10	>1.3 × 10^9^

### Discussion

We began our studies using information gathered in *E. coli *as a reference point. In *E. coli*, *recA*-dependent homologous recombination relies on the RecBCD pathway, the RecFOR pathway (originally designated the RecF pathway) and the RecE pathway [38]. The RecBCD pathway is important in conjugational and transductional recombination [39], and may also be involved in the recombination of plasmids containing one or more Chi sites [40]. Recombination in small plasmids lacking a Chi sequence is primarily catalyzed by the RecFOR pathway [41]. RecF, RecO, and RecR bind to gaps of ssDNA and displace the single-strand DNA binding proteins to allow RecA to bind [42,43]. The RecJ ssDNA exonuclease acts in concert with RecFOR to enlarge the ssDNA region when needed. Strand exchange is then catalyzed by RecA [44]. Because of their prominent role in plasmid recombination in *E. coli*, we analyzed the effect of mutations in *recF*, *recJ *and *recA *on plasmid recombination in *Salmonella*.

Attenuated *S*. Typhi strains have been developed as antigen delivery vectors for human vaccine use. Due to the host restriction phenotype of *S*. Typhi, preliminary work is typically done in *S*. Typhimurium using mice as the model system to work out attenuation and antigen expression strategies. Recently, we have also been investigating attenuated derivatives of the host-restricted strain *S*. Paratyphi A as a human vaccine vector. Therefore, it was of interest to evaluate and compare the effects of *rec *mutations in these three *Salmonella *serovars. We selected *S*. Typhi strain Ty2 as exemplary of this serovar because most of the vaccines tested in clinical trials to date have been derived from this strain [45]. *S*. Typhi strain ISP1820 has also been evaluated in clinical trials [46,47] and we therefore included it in some of our analyses. We found that, for some DNA substrates, the effects of Δ*recA *and Δ*recF *deletion mutations differed among *Salmonella enterica *serotypes. In particular, we found that deleting *recA*, *recF *or *recJ *in *S*. Typhi Ty2 and deleting *recF *in strain ISP1820 had significant effects (3-10 fold) on the recombination frequency of our direct repeat substrate, pYA4463 (Table 3). No or very limited effect (< 2 fold) was observed for our *S*. Typhimurium and *S*. Paratyphi A strains, consistent with results reported for *E. coli *indicating that recombination of this type of substrate is *recA*-independent [35]. In contrast, the Δ*recA *and Δ*recF *mutations resulted in lower interplasmid recombination in Typhimurium and Paratyphi A but not in Typhi strains. Deletion of *recJ *led to a reduction in intraplasmid recombination frequencies in *S*. Typhi, while no effect was seen in *S*. Typhimurium. The Δ*recJ *mutation also affected plasmid recombination frequencies for two of the three substrates tested in *S*. Paratyphi A. Taken together, these results suggest that the recombination system in *S*. Typhi, or at least in strains Ty2 and ISP1820, is not identical to the recombination system in *S*. Typhimurium and *S*. Paratyphi A.

To investigate the mechanism responsible for the observed differences, we analyzed the genome sequences of *S*. Typhimurium UK-1 (Luo, Kong, Golden and Curtiss, unpublished whole genome sequence), *S*. Paratyphi A (NC_006511) [48] and *S*. Typhi Ty2 (NC_004631) [49]. No paralogs of the *recA*, *recF *and *recJ *genes were found in the three strains. The *S*. Typhimurium UK-1 has RecA, RecO and RecR protein sequences identical to Typhi Ty2, and RecF and RecJ protein sequences with over 99% identity. Plasmids expressing Typhimurium *recF *or Typhi *recF *complemented the Δ*recF126 *mutation in Typhi, as evidenced by the UV sensitivity profile (Figure 4) and intraplasmid recombination of pYA4463 (Table 3). Therefore, the basis for these differences are not clear and indicates that there may be other genes or gene products involved. A more detailed analysis of this phenomenon is under investigation.

Plasmid recombination frequencies were higher in our *Salmonella *strains than those reported in *E. coli*. We observed intra- and interplasmid recombination frequencies on the order of 1 × 10^-3 ^in Rec^+ ^*Salmonella*, whereas measurements made in *E. coli *strain AB1157 using a similar plasmid system (equivalent to our substrates pYA4590 and pYA4464 + pYA4465) revealed a basal frequency around 10-fold lower, approximately 1 × 10^-4 ^for both types of substrates [26]. Interestingly, the effect of a *recF *mutation in *E. coli *was to reduce the recombination frequency of intra- and interplasmid recombination approximately 30-fold, to roughly the same frequencies we observed for *S*. Typhimurium (Table 3). However, consistent with the results in *E. coli*, the effects of *recA*, *recF*, and *recA recF *mutations were similar, indicating that the mutations are epistatic.

RecF has been shown previously to play a role in recombinational repair of chromosomal DNA in response to DNA damaging agents [50], including a major role in homologous recombination between direct repeats in the chromosome of *S*. Typhimurium. In our study, we did not observe any effect of *recF *on intrachromosomal recombination, although it did have an effect on the frequency of plasmid integration (Table 4). This discrepancy can be explained by the fact that we did not use DNA damaging agents in our study. These agents lead to single stranded stretches of DNA that represent substrates for *recF *(and *recA*). Our observation that *recF *did affect plasmid integration may reflect the presence of stretches of ssDNA in the plasmid, presumably due to supercoiling effects.

To induce strong primary and memory immune responses, *Salmonella *delivery vectors should be sufficiently invasive and persistent to allow antigen expression in targeting organs, while maintaining a high degree of safety. This requires the use of mutations that attenuate the *Salmonella *vector without impairing its antigen delivery ability. Many attenuating mutations impair invasion and colonization ability. In our study, we confirmed a previous report that *recF *is not required for *S. *Typhimurium virulence in mice [51], indicating that the *recF *mutant remains invasive and replicates well in colonized organs. Therefore, including a Δ*recF *mutation in a *Salmonella *vaccine strain is unlikely to affect its immunogenicity. Our results with the *S. *Typhimurium *ΔrecA *strain are consistent with two previous, independent studies showing that *recA *mutations reduce *Salmonella *virulence [51,52]. To evaluate the potential effect of Δ*recA *mutation on immunogenicity, mice inoculated with the *recA *mutant were challenged with a lethal dose of virulent wild-type *S. *Typhimurium. All the challenged mice survived, indicating that a Δ*recA *mutant retains immunogenicity and therefore may be suitable for use in a vaccine. However, since it does not affect virulence, inclusion of a Δ*recF *mutation into a *Salmonella *vector that has been attenuated by other means to reduce the frequency of intra- and interplasmid recombination, may be more desirable than a Δ*recA *mutation. Studies are currently underway to investigate these possibilities.

Our data show that Δ*recA *and Δ*recF *mutations resulted in reduced frequencies of intraplasmid recombination in all *Salmonella *strains tested, which included three serovars, when there was an intervening sequence between the direct duplications (Table 3). Our results also show that it is likely that deletions in *recA*, *recF *or *recJ *will not be useful for reducing interplasmid recombination in *S. *Typhi vaccine strains, since we did not observe any reduction in interplasmid recombination frequency. This result was disappointing, since the majority of human trials with live *Salmonella *vaccines have focused on *S*. Typhi. In the case of *S*. Typhi, it appears that the best approach to preventing interplasmid recombination will be in the careful design of each plasmid, avoiding any stretches of homology. However, for vaccines based on *S*. Typhimurium or *S*. Paratyphi A, introduction of a Δ*recF *mutation into attenuated *Salmonella *vaccine strains carrying multiple plasmids is a useful approach to reduce unwanted plasmid/plasmid or plasmid/chromosome recombination without further attenuating the strain or negatively influencing its immunogenicity. The Δ*recA *mutation had a similar or more pronounced effect on reducing various classes of recombination and it clearly had an effect on virulence. We did not examine the effect of a Δ*recA *mutation on the immunogenicity of a vectored antigen. Based on its effect on virulence, it may affect the immunogenicity of the vectored antigen in some attenuation backgrounds and therefore may not be applicable for all attenuation strategies.

## Conclusions

In this study we showed that Δ*recA *and Δ*recF *mutations reduce intraplasmid recombination in *S*. Typhimurium, *S*. Typhi and *S*. Paratyphi while there is an intervening sequence between the duplicated sequences. The Δ*recA *and Δ*recF *mutations reduce interplasmid recombination in *S*. Typhimurium and *S*. Paratyphi but not in *S*. Typhi. The Δ*recF *mutations also sharply reduce intraplasmid recombination between direct duplications in *S*. Typhi. Since Δ*recA *mutation results in an avirulent *Salmonella *strain, the Δ*recF *mutation is ideal for reducing plasmid recombination in *Salmonella *delivery vectors without impairing the virulence. The intrachromosomal recombination and plasmid integration are 2-3 orders lower than plasmid recombination, therefore are less concerned. These information help develop *Salmonella *delivery vectors able to stably maintain plasmid cargoes for vaccine development and gene therapy.

## Methods

### Bacterial strains and media

*E. coli *K-12 strain EPI300™ was used for cloning and stable maintenance of plasmids. All *Salmonella *strains used in this work were derived from *Salmonella enterica *serovar Typhimurium wild-type (wt) strain χ3761 (UK-1), serovar Typhi strains Ty2 and ISP1820 or serovar Paratyphi A strain χ8387. Their origin and relevant genotypes are presented in Table 2. Bacteria were grown in LB broth [53].

### Plasmid construction

All plasmids used in this study and their relevant characteristics are presented in Table 1. Primers used for plasmid construction are shown in Table 6. All enzymes were obtained from New England Biolabs or Promega.

**Table 6:** Primers used in this study

Primer	Sequence^a^	Direction^b^
P1	tatttctagatttcagtgcaat	F
P2	ttaggtaccgcgaacgccagcaagacg	R
P3	taaggtaccccggaattgccagctggg	F
P4	ttaggatcctccgcgcacatttccccg	R
P5	taacccgggaattctcatgtttgac	F
P6	ttaagatctccatgccggcgataat	R
P7	tgcttcaacagtacgaattcactatccggttcaataccaagttgcatgacgcatgcctgcagggcgcg	F
P8	gttttgctgaatggcggcttcgttttgcccgccccaccatcacctgatgattatttgttaactgttaattgtc	R
P9	ggcaacaatttctacaaaacacttgatactgtatgagcatacagtataattgcttcaacagtacgaa	F
P10	gagaaatgccaaaagggccgcataaatgcagcccttgatggtaatttaacgttttgctgaatggcggc	R
P11	taaactagtacgacagcagagtcctgtaccg	F
P12	ttaggtacctgaagcttgtcatgcaacttggtattgaac	R
P13	taaggtaccggatcctcatcaggtgatggtggggcgg	F
P14	ttatctagatttgcgaacggcctgttcacgt	R
P15	gatagcacgtgctatcttgtgc	F
P16	tcgtcgcagacgctgttcgccg	R
P17	ctagtctagacgtcagtgagaatcagctcaaa	F
P18	caaggtaccatattagtacattcgtccagg	R
P19	cgcggtaccagcgctgaacacgttatagacat	F
P20	acatgcatgcgaatagtcacgacgatatcttt	R
P21	ctagtctagacgtcagtgagaatcagctcaaaatc	F
P22	cggggtaccatcaactcataaccagggcgttatc	R
P23	cgactttatctttacctcgaagctggtggat	F
P24	gttacggacacggagttatcggcgtgaata	R
P25	ctagtctagaagattataacgcgctggg	F
P26	cggggtaccgcgtattatttaccactggtc	R
P27	cgcggtacctaatcggggcgatttaacaac	F
P28	acatgcatgccttcgagcgatgaacgctct	R
P29	gtctataaagcgccggatgagaaacatgtc	F
P30	tcgacgatcgcttcgagcgatgaacgctct	R
P31	taaaagcttgaccgcgactgtctgatcgt	F
P32	tcaagatctctcgggcgcggagttgcccggc	R
P33	taaagatcttgactgcagtgaaaaagcagtttgccacgat	F
P34	ttagagctcagaaaggaataccggcatgaca	R
P35	taaagatctcgatataagttgtaattctc	F
P36	ttactgcaggcgaggtgccgccggcttcc	R
P37	ttactgcagtccgcgcacatttccccg	R
P38	ggggtaatgtcgtggaccatttgc	F
P39	ccgcggtaatccccggcactaccg	R
P40	gcgctacaaaccctgtggcaacaat	F
P41	gctgtgatcgcggacagcaagaatac	R
P42	ttctcaacataaaaaagtttgtgtaatactgaggatgcggcgtcacag	F
P43	gttacggacacggagttatcggcgtgaata	R

^a ^The underlined sequences are enzyme sites mentioned in the text.

^b ^Forward (F) or reverse (R) primers.

To construct plasmid pYA4463 (Figure 1 panel A), a *Xba*I-*Hinc*II fragment containing the *tetA *promoter and 568 bp of the 5' end of *tetA*, was excised from pACYC184 and ligated into *Xba*I-*Eco*RV digested pACYC184.

To generate plasmid pYA4590 (Figure 1 panel A), the 5' end of *tetA *gene together with its promoter was amplified from pACYC184 with primers P1 and P2, which contain engineered *Xba*I and *Kpn*I restriction sites, respectively. The resulting PCR fragment was digested with *Xba*I and *Kpn*I. The *kan *gene was amplified from plasmid p15A-PB2-kan, a pACYC184 derivative carrying a influenza virus PB2 gene and a *kan *cassette, with primers P3 and P4, which were engineered to contain *Kpn*I and *Bam*HI sites, respectively. The resulting PCR fragment was digested with *Kpn*I and *Bam*HI. The two digested PCR fragments were ligated into pACYC184 digested with *Xba*I and *Bam*HI. The resulting plasmid, pYA4590, contains the *tetA *promoter and 891 bp of the 5' end of *tetA*, a 1041-bp fragment encoding *kan *and its promoter followed by 902 bp of the 3'end of *tetA*.

To construct plasmid pYA4464 (Figure 1 panel B), plasmid pACYC184 was digested with *Xba*I and *Eco*RV to remove the 5' 102 bp of the *tetA *gene and the *tetA *promoter. The cohesive ends were filled using the Klenow large fragment of DNA polymerase and the linear plasmid was self-ligated to yield plasmid pYA4464.

To construct plasmid pYA4465 (Figure 1 panel B), the 5' 853 bp of *tetA *together with its promoter was amplified from pACYC184 using primers P5 and P6, which were engineered with *Sma*I and *Bgl*II sites, respectively. The resulting PCR fragment was digested with *Sma*I and *Bgl*II, and ligated to *Eco*RV and *Bgl*II digested pBAD-HisA.

### Creation of *rec *deletions

The *recA62 *deletion, which deletes 1062 bp, encompassing the entire *recA *open reading frame, introduced into the bacterial chromosome using either λ Red recombinase-mediated recombination [54], or conjugation with *E. coli *strain χ7213(pYA4680) followed by selection/counterselection with chloramphenicol and sucrose, respectively [55]. The *cat*-*sacB *cassette was amplified from plasmid pYA4373 by PCR with primers P7 and P8 to add flanking sequence. The PCR product was further amplified with primer P9 and P10 to extend the flanking sequence. Those two steps of amplification resulted in the *cat*-*sacB *cassette flanked by 100 bp of *recA *flanking sequences at both ends. The PCR product was purified with QIAquick Gel Extraction Kit (QIAGEN) and electroporated into *Salmonella *strains carrying plasmid pKD46 to facilitate replacement of the *recA *gene with the *cat*-*sacB *cassette. Electroporants containing the *cat-sacB *cassette were selected on LB plates containing 12.5 μg chloramphenicol ml^-1^. From *S*. Typhimurium chromosome, a 500-bp sequence upstream *recA *gene was amplified with primers P11 and primer P12 and a 500-bp sequence downstream *recA *gene was amplified with primers P13 and P14. Primers P12 and P13 were engineered with a *Kpn*I site. The two PCR fragments were digested with *Kpn*I, ligated and amplified with primers P11 and P14. The resulting PCR product was digested with isocaudarner *Spe*I and *Xba*I and ligated into *Xba*I-digested pRE112 to yield plasmid pYA4680. In addition, undigested, agarose-gel purified PCR product was electroporated into the *cat*-*sacB Salmonella *strains carrying plasmid pKD46 and spread onto LB plates containing 5% sucrose to select for deletion of the *cat*-*sacB *cassette. Chloramphenicol-sensitive isolates were verified as Δ*recA62 *by PCR using primers P15 and P16 (Δ*recA62*: 1360 bp; wt: 2412 bp). *S*. Typhimurium strains χ9833 and χ9939 were constructed by this method (Table 2). For construction of a Δ*recA62 *mutant of *S. *Typhi, wild-type strain Ty2 was mated with *E. coli *strain χ7213(pYA4680). Transconjugants were selected on LB plates containing chloramphenicol, followed by counterselection on sucrose plates as described above. The resulting Δ*recA62 *strain was designated χ11159. The *S*. Paratyphi A strain χ11243 was generated from wild-type strain χ8387 using the same strategy.

The Δ*recF *deletion strains were constructed using suicide vectors pYA3886 and pYA4783. From the *S*. Typhimurium chromosome, a 397-bp sequence upstream of the *recF *gene was amplified with primers P17 and P18, which were engineered with *Xba*I and *Kpn*I sites, respectively. The downstream 296-bp sequence (including 78 bp from the 3' ORF of *recF*) was amplified with primers P19 and P20 containing *Kpn*I and *Sph*I sites, respectively. The two fragments were digested and inserted into *Xba*I-*Sph*I digested pRE112, resulting in plasmid pYA3886. The corresponding deletion was designated Δ*recF126*. Strains χ9070, χ9081 and χ11244 were generated by conjugation using *E. coli *strain χ7213(pYA3886). Phage P22HT*int *mediated transduction was used to construct Typhi strain χ11053 [56]. The Δ*recF126 *deleted 996 bp from the 5'end of *recF *in serovars Typhimurium and Paratyphi. The upstream flanking sequence of *S*. Typhi is different with the other serotypes. To construct a serovar Typhi-specific Δ*recF *mutation, we constructed a new suicide vector. The *recF *upstream flanking sequence in plasmid pYA3886 was replaced with the corresponding DNA sequence (447 bp) from *S*. Typhi Ty2. Primers P21 and P22 were used for this modification. The resulting plasmid was designated as pYA4783. The Typhi-specific Δ*recF1074 *mutation was introduced into *S*. Typhi strains ISP1820 and Ty2 by conjugation with *E. coli *strain χ7213(pYA4783) to yield strains χ11133 and χ11134, respectively. Primers P23 and P24 were used to verify the *recF126 *and *recF1074 *deletions.

Similar strategies were used to construct the Δ *recJ1315 *deletion with suicide vector pYA3887. From the *S*. Typhimurium chromosome, 330 bp upstream of the *recJ *gene was amplified with primers P25 and P26, which were engineered with *Xba*I and *Kpn*I sites, respectively. The 299-bp downstream sequence was amplified with primers P27 and P28, engineered with *Kpn*I and *Sph*I sites, respectively. The two fragments were digested and ligated with *Xba*I-*Sph*I digested pRE112. The resulting plasmid was designated pYA3887 and the corresponding deletion was named Δ*recJ1315*. Strains χ9072 and χ11245 were generated by conjugating the parental strains with *E. coli *strain χ7213(pYA3887). Strain χ11194 was constructed by phage P22HT*int *mediated transduction. The Δ*recJ1315 *mutation is a deletion of the entire *recJ *gene (1734 bp). Primers P29 and P30 were used to verify the *recJ1315 *deletion (Δ*recJ1315*: 736 bp; wt: 2461 bp).

To test chromosome-related recombination, the 5'*tet *and 3'*tet *fragments were inserted into the *cysG *gene of each *S. *Typhimurium strain using the λ Red system. The 460-bp fragment of the *cysG *gene was amplified using primers P31 and P32 that were engineered with *Hind*III and *Bgl*II sites, respectively. The PCR product was digested with *Hind*III and *Bgl*II. A 480 bp adjoining fragment of *cysG *was amplified with primers P33 and P34. Primer P33 was engineered with *Bgl*II and *Pst*I sites and primer P34 was engineered with a *Sac*I site. The PCR product was digested with *Bgl*II and *Sac*I. The two digested PCR fragments were ligated into *Hind*III and *Sac*I digested pYA4518, deleting green fluorescent protein (GFP) gene. The resulting plasmid pYA4518-cysG has *Bss*HII and *Pst*I sites between the two *cysG*-fragments. This plasmid was digested with *Bss*HII, followed by treatment with the Klenow large fragment. The linear plasmid was further digested with *Pst*I for insertion of truncated *tetA *genes. The 5'*tet-kan*-3'*tet *cassette was amplified from pYA4590 with primers P35 and P36. Primer P36 was engineered with a *Pst*I site. The PCR product was digested with *Pst*I and inserted between the *cysG *fragments in pYA4518-cysG to yield plasmid pYA4689. The 5'*tet-kan *cassette was amplified from pYA4590 with primers P35 and P37. Primer P37 was engineered with a *Pst*I site. The PCR product was digested with *Pst*I and inserted into treated pYA4518-cysG to obtain plasmid pYA4690. The 5'*tet-kan*-3'*tet *cassette, together with *cysG *flanking sequences, was amplified from pYA4689 using primers P31 and P34. The PCR product was electroporated into strains χ3761(pKD46), χ9070(pKD46), χ9072(pKD46) and χ9833(pKD46) with selection on LB plates containing 25 μg/ml chloramphenicol. After growth at 37°C to cure plasmid pKD46, the resulting strains containing chromosomal copies of the 5'*tet-kan*-3'*tet *cassette in *cysG *were designated χ9931 (Rec^+^), χ9932 (Δ*recF*), χ9933 (Δ*recJ*) and χ9934 (Δ*recA*), respectively. Primers P38 and P39 were used to verify insertion in the *cysG *gene. The 5'*tet-kan *cassette together with *cysG *flanking sequences was amplified from pYA4690 with primers P31 and P34. Using the same strategy, the PCR product was electroporated into pKD46 transformants of strains χ3761, χ9070, χ9072 and χ9833 to yield strains χ9935 (Rec^+^), χ9936 (Δ*recF*), χ9937 (Δ*recJ*) and χ9938 (Δ*recA*), respectively, each containing the 5'*tet-kan *cassette inserted into *cysG*. These strains were transformed with plasmid pYA4464 to test plasmid integration based on the 789-bp of *tetA *sequence common to both the plasmid and the bacterial chromosome.

### Analysis of recombination frequency

To examine plasmid recombination and plasmid integration, plasmid(s) containing truncated *tetA *genes were introduced into *Salmonella *strains with or without *rec *mutations. The resulting strains were inoculated into 3 ml of LB broth supplemented with 100 μg/ml ampicillin and/or 25 μg/ml chloramphenicol, as needed. After 8 h growth at 37°C, bacteria were serially diluted in 10-fold steps. 100 μl of the 10^-2^, 10^-3 ^or 10^-4 ^dilution were spread onto LB-agar plates supplemented with 10 μg tetracycline ml^-1 ^and 100 μl of the 10^-5^, 10^-6 ^or 10^-7 ^dilutions were spread onto LB-agar plates with or without the addition of antibiotics, as needed. Plates were incubated overnight at 37°C. The ratio of tetracycline resistant colonies to total colonies was calculated as the recombination frequency. The average mean frequency was calculated using the frequencies obtained from 3-10 assays for each strain. Following one-way ANOVA, the Dunnett's test was used to compare multiple groups against the control. The Student's *t*-test was used to analyze two independent samples.

### Complementation of *rec *mutation

Plasmid pYA5001 has a pSC101 *ori*, a gentamicin resistance marker and a prokaryotic green fluorescent protein (GFP) gene cassette flanked by two *Ahd*I sites. A linearized T vector for cloning PCR products can be obtained by removing the GFP cassette by *Ahd*I digestion. The *recA *genes from *S*. Typhimurium and *S*. Typhi were amplified using their respective chromosomal DNAs as template with primers P40 and P41. The *recF *genes were amplified similarly using primers P42 and P43. The forward primer P42 was engineered to include the *S*. Typhimurium *lpp *promoter sequence ttctcaacataaaaaagtttgtgtaatact (the -35 and -10 boxes are underlined). Amplified DNA fragment were treated with Taq DNA polymerase in the presence of dATP to add 3' A overhangs. Then the treated PCR products were cloned into pYA5001-derived T vector to yield *recA *plasmids pYA5002 (Typhimurium) and pYA5004 (Typhi), and *recF *plasmids pYA5005 (Typhimurium) and pYA5006 (Typhi). The *recA *plasmids, *recF *plasmids or empty vector plasmid pYA5001 were transformed into *S. *Typhimurium *recA *or *recF *mutants, respectively for complementation studies. The *recA *and *recF *plasmids were also introduced into *Salmonella *strains carrying pYA4590 or pYA4463 to complement the *rec *mutation and measure the plasmid recombination frequency.

### UV sensitivity test

Quantitative UV killing curves were measured as described previously [57]. Briefly, cells were grown in 3 ml of LB broth at 37°C with vigorous shaking to mid-log phase. The cells were then 10 fold serially diluted in buffered saline with gelatin (BSG) and spread on LB agar plates. Multiple dilutions were exposed to 254 nm UV in a dark room at each designated dose. Then the plates were wrapped with aluminum foil and placed at 37°C overnight. The 10^-6 ^dilutions were not exposed to UV to determine the total bacterial cell numbers present in the culture. Surviving fractions were calculated as the CFU remaining after UV exposure/total CFU present.

### Virulence determination of the *rec *mutants

Eight-week old BALB/c female mice were purchased from Charles River Laboratories (Wilmington, MA). Mice were held in quarantine for 1 week before use in experiments. Food and water were deprived 6 h before administration of bacteria. Each mouse was orally inoculated with 20 μl of *Salmonella *suspended in buffered saline with gelatin (BSG) by pipet feeding. Food and water were returned 30 min after inoculation. All mice were observed for a month to record mortality. The 50% lethal dose (LD_50_) was determined via the Reed and Muench method [58]. Surviving mice were challenged orally with wild-type *Salmonella *χ3761 two months after the first inoculation.

## Competing interests

The authors declare that they have no competing interests.

## Authors' contributions

RC, XMZ and WK conceived and designed the study. XMZ, SYW and KB constructed plasmids and *Salmonella *strains. XMZ performed all DNA recombination assays. XMZ, WK and XZ carried out the animal experiment. XMZ and KR performed UV killing experiment and wrote the manuscript. All authors read and approved the final manuscript.
